# Social norms and fertility intentions: Evidence from China

**DOI:** 10.3389/fpsyg.2022.947134

**Published:** 2022-11-17

**Authors:** Xiao Yu, Jianing Liang

**Affiliations:** ^1^Northeast Asian Research Center, Jilin University, Changchun, China; ^2^Northeast Asian Studies College, Jilin University, Changchun, China

**Keywords:** social norms, neighborhood social norms, group social norms, fertility intentions, China

## Abstract

China’s low fertility rates are a major concern across all sectors of society. Fertility is a major issue related to economy, society and family development. Based on social norms theory, this paper explores the influence of social norms on individuals’ fertility intentions from two perspectives: spatial proximity and social proximity. Using data from the China Family Panel Studies, we found that individual’s fertility intentions were influenced by social norms; both neighborhood social norms and group social norms had significant effects. The role of social norms in shaping individual fertility intentions varied by gender, *hukou*, and life course; specifically, men, rural residents, and married individuals were more significantly influenced by social norms. This study improves the theoretical framework of fertility decision making by arguing that in addition to macro and individual factors, social norms have a very important influence on fertility intentions. Our findings suggest that reshaping social norms regarding fertility is essential to enhance fertility rates in China.

## Introduction

**Figure 1:**
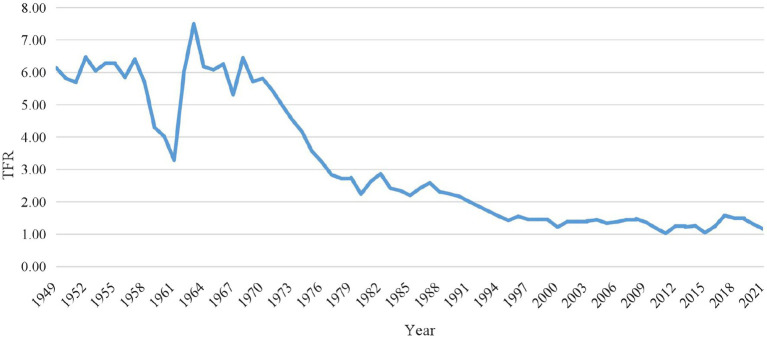
China’s TFR, 1949–2021.

Changes in population development dynamics affect the prospects of China’s economic and social development. Since the founding of the country, China’s fertility rate has fluctuated greatly (see Figure 1). Along with its implementation of the One-Child Policy and modernization development, China’s fertility rate has swiftly declined, and a demographic transition has occurred. In 1990, China’s total fertility rate (TFR) fell to a generation-replacement level of 2.1 and has continued to decline yearly, reaching 1.5 after 2000; thus, the country has entered a phase of long-term low fertility. To alleviate the structural problems brought about by its low fertility rate (such as decrease in working age population and population aging), China began to adjust its family planning policies and implemented a comprehensive the Two-Child Policy in 2016. There was a brief rebound in births in 2016 when the number of births reached 17.86 million (Statistic Bureau of the People’s Republic of China, 2020). However, the rebound in fertility levels under the policy did not last, and the number of births declined yearly 2017 onwards (Statistic Bureau of the People’s Republic of China, 2020). Data from the Seventh National Census indicate that China had only 12 million births in 2020, while TFR reached 1.3 and the country entered an ultra-low fertility stage (Beijing News, 2021). To improve its demographic structure and implement a national strategy to actively cope with population aging, China amended the Law of the People’s Republic of China on Population and Family Planning in August 2021, thereby allowing couples to have three children. The law provides relevant support measures to reduce the burden of fertility, child rearing and education, and aims to create a social environment that is conducive to fertility and to gradually increase fertility rates. However, the Three-Child Policy has not achieve the desired effect, with only 10.62 million births in 2021 and TFR of only 1.15. China has continued to be at ultra-low fertility levels (Liang, 2022).

Scholars have richly explored the causes of low fertility levels in China, offering explanations in terms of modernization and its associates [e.g., economic development (Wang and Chi, 2017; Yu et al., 2021), rising female educational (Zhao, 2019; Chen et al., 2022), health (Clark et al., 2020; Zhang, 2020)], fertility policies (Jia, 2009; Zeng and Hesketh, 2016; Zhang, 2017), and individual factors (Wei et al., 2018; Shen et al., 2020; Wang and Qiao, 2020; Zhang et al., 2022), respectively. Most of these studies have been analyzed in the framework of economic, wherein the decision to have children is examined based on cost–benefit analyses. However, human beings are complex beings, while economics only constitute a fraction of individuals’ reality. Humans’ behavior and decision-making are not determined by its own factors, but are embedded in a particular sociocultural environment (Takac et al., 2011; Qin, 2017). Accordingly, individual behavioral attitudes and decisions are not only rational judgments based on individual preferences and constraints, but are also influenced by the social environment, social opinion, social norms, group pressure, and other factors (Ryu and Jang, 2006; Cislaghi and Shakya, 2018). Social norms influence individual behavioral attitudes and values in several areas, such as pro-environmental behavior (Farrow et al., 2017), age of marriage (Kenny et al., 2019), women’s labor participation (Codazzi et al., 2018), health (Cislaghi and Shakya, 2018) and consumption decisions (Pristl et al., 2021). The individual fertility decision is not only affected by the individual characteristics, but also by the social norms of the reference group, which has recently become a topic of interest for sociologists and demographers. The desire to have a child, the timing of childbirth and available support are all influenced by social norms (Rossier and Bernardi, 2009), which has been observed in many national contexts, for example, India (Jayakody et al., 2008; Mishra and Parasnis, 2017), Bangladesh (Munshi and Myaux, 2006; Rabbi, 2014; Nahar and Zahangir, 2019), Sub-Saharan countries (Barrett et al., 2020), Costa Rica (Rosero-Bixby and Casterline, 1994; Lima et al., 2018; Blanco, 2019), and the United States (Axinn et al., 1994; Ciliberto et al., 2016; Beaujouan and Berghammer, 2019). Differential pattern theory suggests that China is a “acquaintance society” based on ties of kinship, blood, and geography, with its own unique trust system and social norms (Fei, 1998). Compared with other countries, in Chinese acquaintance society, social norms have a more profound influence on individuals’ behavioral attitudes (Fei, 1998; Hofstede, 2001; Wang et al., 2022). However, to what extent is an individual’s conception of fertility affected by social norms in the Chinese cultural context?

Chinese fertility culture (strongly influenced by the patriarchal system and agrarian society) could be summarized with “many children, many blessings.” This ensured the survival of Chinese civilization against the background of high mortality rates. This traditional fertility culture lasted for thousands of years and was engrained as a social norm among the Chinese, thereby influencing individual’s family planning. After the founding of the People’s Republic of China (PRC), this fertility culture remained. Except for a brief decline due to external factors such as China’s Great Famine, China’s TFR remained high (around 5 or 6) from 1949 to the 1970s. However, the improvement of medical conditions and living standards after the founding of the PRC led to a rapid decline in mortality. This high birth rate, coupled with lower mortality rates, caused the rapid growth of China’s population. To alleviate the contradiction among population development, environmental capacity, and socioeconomic development, China began to implement the One-Child Policy (OCP). However, traditional fertility norms are inertial and persistent, resulting in a small drop in fertility when the OCP was first decreed (Feng, 2021). Subsequently, with the OCP and further economic and social development, fertility behaviors and attitudes gradually began to change. Social norms have shifted from “more children, more happiness” to “fewer and better births, more quality.” Studies have shown that having two children has become the ideal family size in the minds of most people (Nie et al., 2021). The social norms have lasting impact on individuals’ fertility intentions and behaviors. Even if the fertility policy has been changed, the social norms would maintain certain inertia and have continuous influence on individual fertility behavior. Therefore, after the implementation of the Three-Child Policy, people still abide by the existing fertility norms of having fewer children. Consequently, fertility levels did not increase rapidly after the Three-Child Policy.

Based on the aforementioned analysis, this paper explores the influence of social norms on individual fertility intentions from two perspectives: spatial proximity and social proximity. This allows us to deepen our understanding of the causes of low fertility rates in China. The contributions of this paper are threefold: first, this study improved the theoretical framework of fertility decision-making, as social norms are the bridge between micro-and macro-factors that affect fertility intentions. Second, most existing studies have focused on explaining fertility from an economic perspective (Wei et al., 2018; Zhao, 2019; Shen et al., 2020; Wang and Qiao, 2020; Chen et al., 2022; Zhang et al., 2022), thus failing to acknowledge human beings as social beings. The individual fertility decision is affected by the social norms of the reference group. The social norms of fertility serve as an informal institution that guides and limits individual decision making. This paper complements the research on social norms on individual fertility from a sociological psychology perspective. The exploration of this issue can help researchers comprehensively understand the causes, persistence, and spread of low fertility levels. Third, clarifying the impact of social norms on individual fertility decisions is also useful for understanding regional and group fertility intentions divergence. This paper has clear policy implications, as family planning policies have not only changed people’s fertility behavior, but also fertility concepts, thereby shaping the social norm for fewer and better births. We must be aware that fertility attitudes and norms are highly inertial and lag significantly behind changes in fertility policy; thus, there is a clear contradiction between existing social norms on fertility and active fertility policy. Therefore, this study highlights that the direction of future policy should be not only to improve fertility support measures, but also to rebuild new fertility social norms and fertility culture, create a fertility-friendly social environment, and change fertility conception of people to improve China’s fertility rate.

## Theoretical review and research progress

### The second demographic transition

The demographic transition (DT) refers to the process of population change from high to low birth and death rates. This demographic transition was based on industrialization and modernization, and the development of economic and medical technology has led to a decline in mortality and an increase in infant survival (Bongaarts and Watkins, 1996; Kirk, 1996). However, DT does not explain the dramatic changes in the population and families that have occurred in Western countries since the 1960s and 1970s, such as below-replacement birth rates, high divorce rates, high cohabitation rates, and significant increases in out-of-wedlock births. In the face of these new changes, demographers developed the second demographic transition (SDT) to explain why fertility has remained below the replacement level (Van De Kaa, 1987).

The social drivers of SDT were not the same as those of demographic transition. SDT suggests that the driving mechanisms behind low fertility are structural social changes, cultural transmutations, and technological innovation. Among these, cultural transmutations are central driving forces. Cultural values occupy a central position in SDT and are intrinsic drivers of changes in marital behavior. SDT argues that the rise of individualism and feminism, gender equality in society, and changes in the concepts of marriage and family are important causes of low fertility intentions and low fertility in postmodern societies (Lesthaeghe, 2010, 2014; Zaidi and Morgan, 2017).

### Gender revolution

Gender revolution is also essential for explaining generally low intentions to marry and fertility. In the first stage of the gender revolution, the increase in women’s education and labor force participation rates broke the previous social structure of the public and private spheres and the gendered division of labor. Women objectively took on more economic functions and subjectively no longer wanted to be confined to the role of the housewife. However, the entrenchment of established gender norms and institutions has led to more severe work—family conflicts for women, which, in turn, has reduced their intentions to marry and have children, leading to a steady decline in marriage and fertility rates (Goldscheider et al., 2015; Raybould and Sear, 2021).

In the second phase of the gender revolution, women’s share of the labor market continues to rise, and gender equality in the public sphere is no longer the only gender revolutionary pursuit for women. Thus, a force for change in the family sphere has developed, and pressure has begun to mount on men to increase their home caring. Driven by a more egalitarian gender perspective, men enter the private sphere more actively and take on family responsibilities, thus contributing to a new equilibrium between stable marriages and rising fertility rates (Goldscheider et al., 2015).

However, there is more resistance to men entering the private sphere than women entering the public sphere. In many countries, the second part of the gender revolution still lags after women have entered the labor market (England, 2010; Sullivan et al., 2018). Women are still the main actors in family affairs, leading to work–family conflict. The stagnation of the gender revolution has caused a decline in fertility intentions.

### Social norms theory

Social Norms are standards of behavior that are widely accepted by members of a group in a given situation (Bicchieri, 2016; Cislaghi and Heise, 2018). They also serve as environmental factors that influence human behavior through sanctions, rewards, and group identity (Storey and Kaggwa, 2009). Social norms are informal institutions that have a restraining effect on people’s behavior (Akerlof, 1980). Unlike explicitly defined rules and regulations, social norms are unwritten and non-mandatory, and their impact is widespread and lasting. Behavioral economists point out that social norms can be internalized into individual consciousness and influence people’s behavior (Zheng et al., 2017). Social norms are considered useful additions or even alternatives to explaining economic behavior beyond “economic rationality” and utility maximization (Elster, 1989). The role of social norms is manifested in many ways, such as pro-environmental behavior (Farrow et al., 2017), women’s labor participation (Codazzi et al., 2018), age of marriage (Kenny et al., 2019), health (Reid et al., 2010; Cislaghi and Heise, 2019), smoking (Garoupa, 2003; Hunter et al., 2020), consumption behavior (Pristl et al., 2021; Melnyk et al., 2022).

During the fertility transition, sociologists and demographers began to focus on the influence of social norms on fertility decisions. Since the 1980s, social interactions and perceptual factors have been used to explain the decline and spread of fertility in Europe (Jayakody et al., 2008; Bernardi and Klärner, 2014). The role of social norms in the demographic transition including the acceptability of national family planning, the spread of modern contraceptive methods (Herbert, 2015; Costenbader et al., 2017), and the rhythm and number of births (Jayakody et al., 2008; Mishra and Parasnis, 2017). Social networks serve as important channels for transmitting social norms, and individuals’ fertility decisions are influenced by important members of their social network. The likelihood that a person will have a child increases significantly within 2 years after a sibling has a child (Kuziemko, 2006). Fertility behavior is significantly “contagious” among friends and colleagues (Hensvik and Nilsson, 2010; Nie et al., 2021). The role of social norms also explains the trend toward smaller ideal family sizes and the increase in childless families in Western countries (Goldstein et al., 2003; Blackstone and Stewart, 2012). Social norms may also affect individual fertility decisions by indirectly influencing the degree of labor force participation (Bekken, 2019; Ciobanu et al., 2019; Wingard, 2019; Pocol et al., 2022).

### Fertility research in China

Looking back at the history of China’s population development (see Figure 1), the TFR fluctuated around a high level in the 1960s. After the 1970s, influenced by multiple factors such as family planning policies and modernization, the TFR declined significantly and had fallen below replacement level in the early 1990s. The TFR declined to 1.5 in 2000 and has remained at a low fertility level since then. China has entered a phase of prolonged low fertility. Changes in China’s fertility levels and fertility social norms can be explained in three ways.

#### Modernization

The first is modernization. Based on DT, industrialization and modernization are believed to lead to a decline in fertility intentions (Yu et al., 2021). Improvements in education levels, health status, and the promotion of women’s employment and status brought about by modernization will all significantly impact fertility intentions. The effect of education on fertility intentions is complex, with access to higher education reducing fertility through channels such as increased investment in children (Zhang et al., 2021), higher childcare costs, changes in traditional fertility attitudes, and delayed age at marriage (Zhou, 2018; Chen et al., 2022). The rise in female status negatively affects fertility intentions because traditional gender roles place significant pressure on women to raise children, resulting in higher opportunity costs for women to have children, including punitive effects on career advancement and wage earnings (Wang and Yang, 2017; Doren, 2019). Becker (1991) referred to children as “household durable” and argued that family fertility intentions increased as household income rises.

#### Fertility policy

The second is the fertility policy. In the early 1970s, the promotion of “late marriage and late childbearing” and “fewer and better births” guided individuals to change from having more children to having fewer. The TFR dropped from 5.81 in 1970 to 2.75 in 1979. In 1980, China formally implemented a family planning policy with the “One-Child Policy” as the main content. Under the combined effect of strict family planning policy and economic development, China’s TFR declined from approximately 2 to <1.5 in the 1990s. Since the 21st century, the fertility rate has been very low (Statistic Bureau of the People’s Republic of China, 2020). China began to adjust fertility policy with the implementation of the Selective Two-Child Policy (couples in which one spouse is the only child can have two children) in 2013 and the Universal Two-Child Policy in 2016. The TFR rebounded slightly but did not reach the expected level. In 2021, China implemented the Three-Child Policy. However, the number of births in 2021 is only 10.62 million, giving a TFR of 1.15 (Liang, 2022). China has rapidly achieved its first demographic transition under the combined effects of fertility policies and modernization. Fertility policies have influenced people’s fertility behavior and, more importantly, changed their fertility concepts. The traditional Chinese concept of “having more children” has been transformed into the modern concept of “having fewer and better children” and has become the conscious action of most people (Chen and Sun, 2021). This modern fertility concept profoundly influences the fertility behavior of the new generation. Even though China is adopting an active fertility policy, the existing social norm of “fewer and better children” has become a constraint on people’s fertility intentions, resulting in the limited effect of China’s active fertility policy (Feng, 2021).

#### Demographic transition

The third is the demographic transition. China has completed its first demographic transition, and fertility has remained below the replacement level for a long time. In addition to the influence of population policy and modernization processes, the SDT is also an important mechanism leading to China’s current low fertility rate. China is currently at the beginning of its SDT (Yu and Xie, 2019). Several young individuals are opting for cohabitation as an alternative to marriage, resulting in the continuous postponement of individuals’ ages at first marriage (Yu and Xie, 2017; Yang, 2021). However, unlike in Western countries, the high cohabitation rate has not resulted in increased extramarital births (Foran et al., 2022). Marriage is still necessary for fertility, as out-of-wedlock births are still not socially and family-accepted due to the influence of traditional concepts in China (Yu and Xie, 2019). Consequently, late marriages have delayed female first births, and the consequent reduction in second and higher births. Due to the influence of Confucian culture, the core position of the family in Chinese society has not been shaken, and family relationships are the most intimate. Simultaneously, in China’s “child-first” family model, parents do their best to provide a suitable environment for their children to grow in, including maintaining the integrity of the family. Consequently, the divorce rate in China has not increased significantly and remains low (Yu and Xie, 2019).

Women enjoy a higher social status and gender equality policies are well implemented in China, and the labor force participation rate of women is high. Simultaneously, Confucian culture profoundly influences China, and women take on traditional family service roles in their households. Therefore, Chinese women shoulder the dual responsibility of social work and family care and face a career-fertility dilemma, an essential factor that reduces women’s fertility intentions (Okun and Raz-Yurovich, 2019; Shen, 2020). The second phase of the gender revolution in China has not yet been completed. Chinese women have higher participation rates in politics, public services, and economic activities but have still not achieved true gender equality in comparative gender power (Meng, 2018). In contrast, men are less involved in the domestic sphere (Li, 2020; Xu, 2020).

Through the review of the aforementioned literature, it could be discovered that the majority of these studies analyzed the influence of individual factors and macro-factors on fertility intention from the perspective of economy (Zeng and Hesketh, 2016; Zhang, 2017; Yu et al., 2021). However, there are few studies focusing on the role of meso-factors (i.e., social-level factors) influencing fertility intentions. Social norms, as important informal institutions, have an significant impact on individuals’ perceptions and values (Farrow et al., 2017; Cislaghi and Shakya, 2018). In the context of the gradual optimization of China’s fertility policy, the influence of social norms on fertility cannot be ignored. The analysis of this issue is of great practical importance for understanding the causes of China’s low fertility rate, including regional and group differences. China’s OCP has brought about an unprecedented fertility revolution in the country, changing the concept of fertility prevalent in traditional Chinese culture, and recreating social norms to prioritize low fertility. Most people have acquired a preference for low fertility and deeply identify with the social norm of low fertility (Zeng and Hesketh, 2016). Therefore, when the fertility policy no longer restricts people from having children, the social norms of low fertility created by OCP would continue to influence people’s fertility intentions and behaviors. Because fertility social norms are persistent and path-dependent relative to fertility policy, low-fertility social norms will have a lasting and far-reaching impact on fertility behavior in China.

## Theoretical framework and research hypothesis

### Theoretical framework

**Figure 2:**
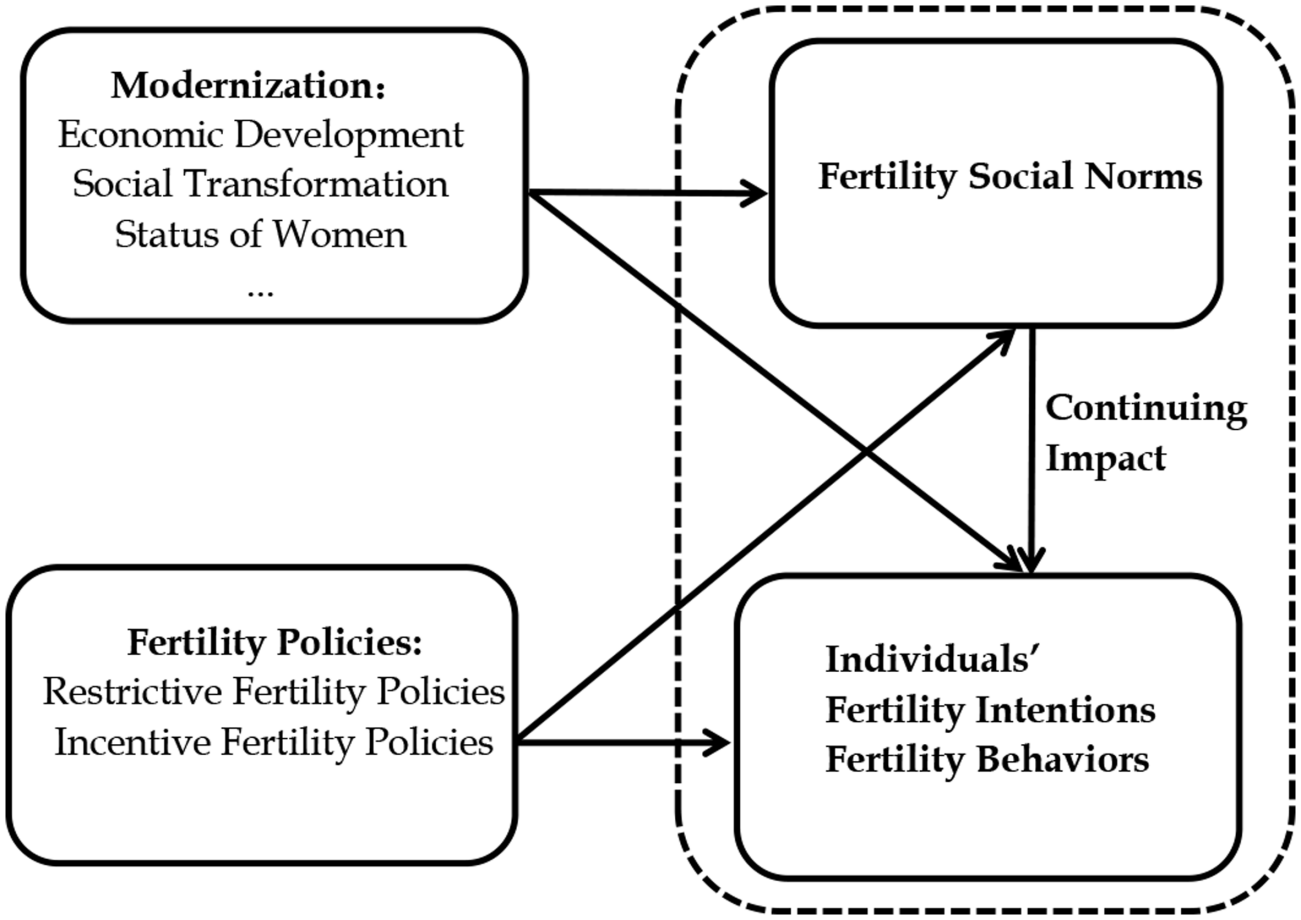
Theoretical framework.

Generally, individuals’ fertility intentions are influenced by two major factors: macro-level modernization, fertility policies and micro-level individual factors (Feng, 2021). Additionally, another critical factor is social norms. We argue that the relationship among these components is that the macro-level modernization process and family policy would, directly and indirectly, impact individuals’ fertility intentions by shaping social norms. Specifically, social norms are a direct result of economic development, social transformation, and demographic transition and a vital mediating variable linking macro-level factors of the country and individual fertility intentions (see Figure 2). On the one hand, fertility policies and modernization processes have directly influenced individual fertility intentions and behaviors, primarily through women’s increased education and social status, and economic independence affecting fertility intentions (Lu and Zhang, 2016; Wang et al., 2017; Jiang, 2020; Chen et al., 2022). On the other hand, these factors also silently shape and change fertility social norms. When new social norms of fertility are formed and are relatively stable, they directly affect individual. Therefore, overall, the fertility intentions and behaviors of individuals result from modernization, fertility policies, individual characteristics, and social norms.

The impact of modernization and fertility policies on the population is not only a change in demographic structure, population development, and population fertility status but also has a more critical connotation of changes in social norms about fertility. While social norms, as informal institutions, do not have as noticeable a constraining effect on individuals’ fertility behavior as formal institutions, relatively flexible social norms may have a more pervasive, deeper, and longer-lasting impact. In particular, social norms maintain inertia when the socioeconomic situation and fertility policies change and continuously influence individual fertility behaviors (Feng, 2021).

The direct effects of macro factors on fertility intentions and the effects of individual characteristics on fertility intentions have been well discussed in the existing literature (Jia, 2009; Wang and Chi, 2017; Zhang, 2017; Yu et al., 2021). However, most of these studies have neglected the effects of macro factors on individual fertility intentions by shaping social norms. Therefore, our study focuses on the effects of social norms on individual fertility intentions (i.e., the dashed line in Figure 2).

According to social norms theory, individuals’ fertility decisions are influenced by the social norms of the reference group (Mishra and Parasnis, 2017). Social norm theory posits that social norms positively impact fertility intention (Lois and Becker, 2014), mainly through social learning, social influence, social pressure, and social support (Rossier and Bernardi, 2009). Social influence means that new ideas an individual receives from others affect their views and values. Individuals change their views and values according to social norms and align with others in the social network to gain the approval of other members and strengthen their sense of belonging and identity. Therefore, the dominant concept of fertility on social networks significantly impacts members (Montgomery and Casterline, 1996). Social learning theory holds that individuals optimize decision-making through social learning, reducing the uncertainty individuals face in decision-making (Umilta et al., 2001). Interactions among social network members can also form a “reference group effect,” wherein a person can be encouraged to make fertility decisions if many of their social network members become pregnant or give birth (Keim et al., 2013). Social pressure means that individuals feel pressure when their behaviors are inconsistent with most members of their social network (Lois and Becker, 2014). They adjust their behaviors to minimize their distance from others, promoting the group’s behavioral consistency. When an individual’s concept of fertility is inconsistent with social norms, social pressure prompts them to adjust their fertility concept to fit social norms (Wu, 2020). Social support means that when an individual follows the existing social norms in the social network, they obtain spiritual, material, and experiential support from those people. In fertility decision-making, when individuals tend to follow social norms, they receive support and help from social network members. The support of social network members for childcare directly affects fertility intention (Bühler and Philipov, 2005).

### Chinese context

China’s cultural context provides a unique case study for examining the relationship between social norms and fertility. First, China has a robust collectivist outlook, and social norms are more influential. Hofstede (2001) notes that China scores higher on collectivist values than many Western countries. The Confucian culture emphasizes harmonious relationships between people, resulting in a strong collectivist cultural environment in Chinese society. Compared to individualistic cultures, people raised in Chinese culture show stronger adherence to the sub-cultural social norms of groups, organizations, and communities and exhibit the same characteristics concerning fertility (Hofstede, 2001; Wang et al., 2022).

Second, China has implemented family planning policies since 1980, which rapidly changed the fertility behavior of residents and shaped the social norm of “fewer and better births.” The fertility concept of “fewer children, better births, later marriage, and later childbearing” is spreading rapidly among the new generation of young people. Simultaneously, the implementation of family planning has led to a rapid increase in women’s educational level, status, and labor force participation rate (Lu and Zhang, 2016; Wang et al., 2017; Jiang, 2020). These factors eventually led to a decline in fertility rates. Thus, China’s family planning policy has shaped new social norms of fertility and triggered a change in fertility levels in society.

Third, significant differences in economic development, social customs, and cultural environments among the Chinese provinces have led to more pronounced regional sub-cultural differences. Therefore, there are also regional differences in fertility culture and attitudes shaped by macro factors (He et al., 2019). For example, in Henan and Shandong, families commonly have two children; however, in Liaoning, Jilin, and Heilongjiang, more families choose to have one child (Statistic Bureau of the People’s Republic of China, 2020). Therefore, studying regional fertility differences in China requires an examination of the impacts of different social norms on fertility.

### Research hypothesis

Individuals develop their fertility preferences by adhering to social norms, which are derived from reference groups in close proximity. These groups can be inherited from the family (family members and religion) and from living communities and workplaces (neighbors and coworkers) (Mishra and Parasnis, 2017). Modern societies are becoming increasingly atomized, and friends and neighbors may be as important as family members. The reasons for this are two-fold: first, declining fertility has led to smaller families, and fewer relatives, friends, and neighbors may have taken the place of siblings; second, friends and neighbors result from individuals’ free choice, and voluntary relationships are more important than family relationships in the SDT (Mishra and Parasnis, 2017; Gao, 2022). This study discusses the impact of social norms outside the family on individual fertility intentions, referring to the existing literature to examine the role of social norms in shaping individual fertility intentions regarding spatial and social proximity (Bongaarts and Watkins, 1996; Tumen and Zeydanli, 2015).

Spatial proximity refers to members living within the same community. Overly large geographic areas or too many people make it challenging to generate communication and cause difficulties in interactions among residents. Thus, an appropriate scope must be chosen to reflect the influence of social norms. Kravdal (2002) points out that the community level provides a suitable measure, with communities or villages characterized by a particular geographic area and number of people, and each community or village has a commonplace and center of activity. Therefore, residents of the same community or village are more likely to have closer social interaction activities and form community social norms in their daily exchanges and communication. Further, they may follow community social norms in choosing their behavior to gain approval (Stinebrickner and Stinebrickner, 2006; Mishra and Parasnis, 2017). Simultaneously, the community serves as a boundary for marking different status groups and reflects the differentiation between different groups.

Therefore, we propose the following hypotheses:

*Hypothesis 1*: Neighborhood social norms based on spatial proximity have a significant effect on individuals’ fertility intentions.

Social proximity refers to members of the same broad occupational group. The reason for using this reference group is that occupational identity is the most important social identity of an individual in social life. The occupational group is formed by different closed mechanisms (e.g., diplomas, certificate licenses, qualification requirements) and has a high level of homogeneity. The nature of the occupation, the content of the work, and social interaction within the occupational group further reinforce occupationally compartmentalized attitudes, values, and lifestyles, which form shared social norms (Li and Zhu, 2017). Different occupational natures and job requirements form different in-group subcultures within occupations (Schein, 1985; Wang et al., 2009). Studies have shown that there are significant differences in fertility intentions among individuals with different occupational backgrounds (Skirbekk, 2008; Wolfinger et al., 2010; Shreffler, 2017). The reason for occupational differences in fertility intentions may be that different occupations have different characteristics (e.g., income and social status), which can lead to differences in the cost of childbearing (Shreffler, 2017; Zeng and Zhao, 2020). Hensvik and Nilsson (2010) study showed that colleagues’ fertility behaviors increase individuals’ likelihood of having children in the short term, validating the influence of intra-occupational social norms and social interactions on fertility from the perspective of short-term fertility decisions.

Therefore, we propose the following hypotheses:

*Hypothesis 2*: Group social norms based on social proximity have a significant effect on individuals’ fertility intentions.

## Data and method

### Data and sample

The data for this study were obtained from 2018 the China Family Panel Studies (CFPS), a national, large-scale, multidisciplinary, and comprehensive social tracking survey that examines changes in China’s society, economy, demographics, education, and health at the individual, household, and community levels. The CFPS adopts the implicit stratification method for multi-stage equal probability sampling; it covers 25 provinces/autonomous regions in China, which account for roughly 95% of the population. The main dependent variable in this paper is fertility intention, which is expressed by the answer to the questionnaire “How many children do you think is ideal for you to have?” The answer to this question for this question ranges from 0 to 10. Based on the distribution of the values of this variable, and to avoid the influence of extreme values, the values of “0” and “1” are combined into “1 or less,” whereas values of “3” or more are combined into “3 or more.”

### Measures

The core independent variable is the fertility social norms. For the measurement of this variable, it is first necessary to specify the reference group, which we define in terms of both spatial proximity and social proximity, according to the aforementioned hypotheses. For spatial proximity, the number of children in the community/ number of people of fertile age in the community (i.e., the actual fertility rate in the community) is calculated and categorized as the “neighborhood social norm.” For social proximity, the classification is based on the respondent’s occupation: farming, business owner, government/ institution/research institute/state enterprise, collective enterprise/ civil non-enterprise/association/community committee, private enterprise/individual business/foreign/Hong Kong, Macao, and Taiwan enterprises/other types of enterprises, and other occupations. The number of children of respondents in a given occupation/number of people of fertile age in a given occupation (i.e., the actual occupational fertility rate) is calculated separately as the group social norm. We use fertility behaviors as proxy for norms because individuals are more likely to observe the former and infer social norms from it. It is difficult for individuals to observe the fertility preferences of others, but it is easy to observe actual fertility behavior (Mishra and Parasnis, 2017). At the same time, to ensure the accuracy of the results, we also conducted regression analysis using the mean of group fertility intentions as a proxy for the specification in the robustness test.

### Control variables

Based on prior research, gender, age, education level, personal income, *hukou*, marital status, ethnicity, health, and siblings were selected as control variables (Wei et al., 2018; He et al., 2019; Yu et al., 2021). As mentioned in 2.4, there are a significant effect of modernization and its associates on fertility intentions. Therefore, we included variables such as economic development of the province, female labor participation rate, industrial structure, urbanization in the control variables. In addition, because the OCP and related incentives are implemented at the provincial level (e.g., the OCP, pension subsidies for one-child parents), provincial-level differences are controlled through provincial fixed effects (Attane, 2002).

The sample we included only the fertile age group. The reasons for this are, first, that even if individuals beyond fertile age have the intention to have children, it is very difficult for them to achieve to it. Studying the fertility intentions of individuals beyond fertile age has limited effect on predicting the future fertility. Second, individuals beyond fertile age may have ended their fertility and their fertility behavior has limited impact on the current individuals of fertile age. Therefore, we mainly examined fertility intention among people under 49 years old as our samples (as a reference, we regressed the full sample, the fertile age sample and the sample beyond fertile age separately in the Appendix). The final sample size was 3,321. The main variable descriptions and descriptive statistics results are shown in Table 1. In the sample, 69.32% of respondents had the intention to have two children, 22.70% intended to have one child or none, and only 7.98% intended to have three children or more. Having two children in a family has become a generally accepted social norm.

**Table 1:** Description of variables and descriptive statistics.

Variables	Variable description	Observations	Mean	Percentage
Fertility intention	Expected number of children	3,321	1.843	
	1 or less	754		22.70%
	2	2,302		69.32%
	3 or more	265		7.98%
Neighborhood social norms	Community actual fertility rate	3,321	1.833	
Group social norms	Occupational actual fertility rate	3,321	1.536	
Gender	Male = 1, Female = 0	3,321	0.531	
		1762		46.94%
		1,559		53.06%
Age	Age of respondents in 2018	3,321	39.15	
Education level	Number of years of education completed	3,321	9.955	
Personal income grouping	Divided into five groups, the lowest is 1 and the highest is 5	3,321	3.066	
Siblings	Number of siblings	3,321	2.276	
*Hukou*	Urban = 0, Rural = 1	3,321	0.676	
	Urban	1,075		32.37%
	Rural	2,246		67.63%
Ethnicity	Han = 1, Minority = 0	3,321	0.925	
	Han	3,072		92.50%
	Minority	249		7.50%
Health	Very healthy = 5, healthy = 4, relatively healthy = 3, general =2, not healthy = 1	3,321	2.836	
Real Estate	No property = 0, have one property = 1, have two or more properties = 2	3,321	1.087	
	No property	323		9.73%
	1 property	2,385		81.54%
	2 or more properties	613		18.46%
GDP		3,321	10.181	
Industrial structure		3,321	0.478	
Female employment rate		3,321	29.935	
Urbanization		3,321	56.993	

Table 2 shows the mean values of fertility intentions by participants from the eastern, central, and western regions in China. The results show differences in fertility intentions among participants from the eastern, central, and western regions. The highest fertility intention is in the western region, followed by the central and eastern regions.

**Table 2:** Distribution of fertility intention: Regional differences.

Region	Observations	Percentage	Mean
Eastern	1,326	39.93%	1.799
Central	1,092	32.89%	1.844
Western	903	27.19%	1.942

Table 3 shows respondents’ fertility intentions within each occupation. The results show differences in fertility intentions within each occupation. Those who work in agriculture and own businesses exhibited higher fertility intentions. Individuals who work for government/institutions/research institutes/state enterprises exhibited the lowest fertility intentions. The reason is that OCP imposes stricter restrictions on them, which makes this group more likely to accept the low fertility patterns (Liu and He, 2016).

**Table 3:** Distribution of fertility intention: Occupational differences.

Occupation	Observations	Percentage	Mean
Farming	536	16.14%	2.017
Business owner	247	7.44%	1.960
Government/institutions/research institutes/state enterprises	673	20.26%	1.786
Collective enterprise/civil non-enterprise/association/community committee	1,539	46.34%	1.801
Private enterprise/individual business/foreign/Hong Kong, Macao, and Taiwan enterprises/other types of enterprises	100	3.01%	1.810
Other	226	6.81%	1.916

### Empirical method

The effect of social norms on individuals’ fertility intentions can be determined as follows:


(1) $$fertility_{i}=\beta_{0}+\beta_{1}norm_{1}+\beta_{2}norm_{2}+\beta_{3}CV_{i}+\varepsilon_{i}$$

where *fertility_i_* denotes the fertility intentions of individual *i*, *norm_1_* denotes neighborhood social norms, *norm_2_* denotes group social norms, and *CV_i_* are control variables and *ε_i_* is random error term. Since the explanatory variable fertility intention is a non-negative discrete random variable, a Poisson regression model should be considered for parameter estimation.

## Results

### Basic results

Table 4 shows the regression results of fertility intention and social norms. The results of model 1 indicate that fertility intentions were significantly positively related to neighborhood social norms at the 1% level, with a regression coefficient of 0.0809. The corresponding marginal effect is 0.1510, indicating that for each unit increase in the community’s actual fertility, the individual’s fertility intention increases by 15.10%. Model 2 examines the relationship between fertility intentions and group social norms. The results show that group social norms had a significant positive effect on individual fertility intentions, with a coefficient of 0.0434, which was significant at the 5% level. The corresponding marginal effect is 0.0810, indicating that for each unit increase in the occupation’s actual fertility, the individual’s fertility intention increases by 8.10%. Model 3 examines neighborhood social norms and group social norms. It shows that both neighborhood social norms and group social norms had significant effect on individual fertility intentions, but the impact of neighborhood social norms on fertility intentions was greater than group social norms.

**Table 4:** Effect of social norms on fertility intention.

Variables	(1)	(2)	(3)
	Fertility intention	Fertility intention	Fertility intention
Neighborhood social norms	0.0809^***^ (7.28)		0.0809^***^ (7.35)
Group social norms		0.0434^**^ (2.42)	0.0431^**^ (2.43)
Gender (reference group: Female)	0.0019 (0.16)	0.0057 (0.48)	0.0040 (0.33)
Age	0.0371^***^ (4.23)	0.0357^***^ (4.04)	0.0355^***^ (4.04)
Age square	−0.0005^***^ (−4.10)	−0.0005^***^ (−4.02)	−0.0005^***^ (−3.91)
Education level	−0.0049^***^ (−2.66)	−0.0067^***^ (−3.60)	−0.0041^**^ (−2.22)
Ethnicity (reference group: Minority)	−0.0958^***^ (−4.29)	−0.0987^***^ (−4.25)	−0.0937^***^ (−4.19)
*Hukou* (reference group: Urban)	0.0131 (0.86)	0.0242 (1.55)	0.0061 (0.39)
Siblings	0.0106^***^ (2.50)	0.0136^***^ (3.16)	0.0102^***^ (2.42)
Personal income grouping	0.0091^*^ (1.83)	0.0074 (1.48)	0.0109^**^ (2.18)
Health	0.0008 (0.15)	0.0003 (0.06)	0.0002 (0.04)
Real estate	0.0284^***^ (2.80)	0.0305^***^ (2.98)	0.0284^***^ (2.80)
GDP	−0.0614 (−0.22)	−0.1441 (−0.50)	−0.0476 (−0.17)
Industrial structure	−0.7859 (−1.47)	−0.6086 (−1.11)	−0.7772 (−1.44)
Female employment rate	−0.0330^**^ (−2.25)	−0.0349^**^ (−2.29)	−0.0355^**^ (−2.43)
Urbanization	−0.0037 (−0.41)	−0.0030 (−0.33)	−0.0029 (−0.32)
Constant	1.2657 (1.01)	2.3624^*^ (1.85)	1.3347 (1.06)
Provincial fixed effect	YES	YES	YES
Observations	3,321	3,321	3,321

*T*-values in parentheses.

* *p* < 0.1;

** *p* < 0.05;

*** *p* < 0.01.

Regarding other factors affecting fertility intention, women are (but not significantly) less likely to have more children. The effect of age on fertility intention shows an inverted U-shape, i.e., the older the individuals is, the higher their fertility intentions; however, after a certain age, fertility intentions gradually decreases. Moreover, the higher the education, the lower the fertility intentions. Individuals with higher education have higher expectations of their career and income, and pay more attention to the realization of their values, and have a higher opportunity cost for having children, that means they are more inclined to have fewer and better children. Han Chinese have lower fertility intentions than other ethnic groups and are more likely to develop low fertility preferences under the long-term implementation of the OCP. Urban residents exhibit lower fertility intentions. The urban and rural areas differences in fertility policies, economic development, and childcare costs lead to the differences in fertility intentions. Additionally, the more siblings an individual has, the higher fertility intention, that comes from the influence of social norms within the family of origin. The increase in personal income eases the financial pressure to have children and boosts fertility confidence. Real estate, as a wealth effect, has a positive effect on fertility intentions.

### Robustness checks

To verify the robustness of the results, we used substitution of core variables and redefinition of fertility intentions to perform robustness tests. First, Ologit, Oprobit, and ordinary least squares (OLS) models were used to analyze the effect of social norms on individuals’ fertility intentions, and the results are shown in Panel A of Table 5. The results using different measures indicated that social norms have significant positive effect on individuals’ fertility intentions, verifying the robustness of the previous results. Second, we used the raw data of fertility intentions without merging and treat them as continuous variables for analysis using the Ols model, as shown in Panel B of Table 5. The robustness of the results is further verified. Third, in the previous section, we used fertility behavior as a proxy. We used fertility intentions as a proxy for robustness testing. The regression results are shown in Panel C of Table 5. The results of all robustness tests were consistent with the previous results, verifying the accuracy of the results.

**Table 5:** Robustness checks.

**Panel A: Change of measurement method**			
	**Ologit**	**Oprobit**	**Ols**
Neighborhood social norm	0.7553^***^ (6.92)	0.4160^***^ (7.19)	0.1596^***^ (7.39)
Group social norms	0.3882^**^ (2.30)	0.2136^**^ (2.37)	0.0805^**^ (2.34)
Control variables	YES	YES	YES
Observations	3,321	3,321	3,321
Provincial fixed effect	YES	YES	YES
**Panel B: Raw data on fertility intention**			
	**Fertility intention**	**Fertility intention**	**Fertility intention**
Neighborhood social norm	0.1004^***^ (7.31)		0.1005^***^ (7.38)
Group social norms		0.0472^**^ (2.23)	0.0470^**^ (2.25)
Control variables	YES	YES	YES
Observations	3,321	3,321	3,321
Provincial fixed effect	YES	YES	YES
**Panel C: Use fertility intention as a proxy**			
Neighborhood social norm	0.1866^**^(6.70)		0.1831^***^(6.58)
Group social norms		0.1137^***^(2.91)	0.0922^**^(2.37)
Control variables	YES	YES	YES
Observations	3,321	3,321	3,321
Provincial fixed effect	YES	YES	YES

*T*-values in parentheses.

** *p* < 0.05;

*** *p* < 0.01.

### Heterogeneity test

#### Urban–rural differences

Under China’s urban–rural dual system, there are significant differences in fertility social norms between urban and rural residents due to differences in fertility policies and socioeconomic environment. Overall, China is a typical acquaintance society, and this characteristic is more prominent in rural areas, where residents are interconnected in a tight social network (Hofstede, 2001; Zeng and Guo, 2016; Wang et al., 2022). Rural residents have smaller social networks and closer social ties, and they tend to make behavioral decisions in consideration of the opinions of their reference groups, and prefer to follow the existing social norms to determine their behavior (Tang et al., 2022). While the frequency of communication between neighbors of urban residents is low (Chai et al., 2017).

Therefore, the samples were divided into urban and rural sub-samples to examine the urban–rural differences in the influence of social norms on fertility intentions. As shown in Table 6, the results showed that both rural and urban individuals’ fertility intentions were significantly positive with neighborhood social norms at the 1% level, indicating that both rural and urban individuals’ fertility intentions were positively influenced by neighborhood social norms. However there were differences in the degree of influence. The coefficient of fertility intentions and neighborhood social norms for the rural sample was 0.0988, higher than that for the urban sample (0.0739). Rural individuals were more influenced by neighborhood social norms. In terms of group social norms, urban individuals’ fertility intentions were significantly and positively related to group social norms, while rural individuals’ coefficient of fertility intentions was positive, but not significant. The reason may be that rural individuals interact more frequently with their neighbors and have closer social ties with neighbors, and are more likely to feel pressure from neighborhood social norms, which could make them more inclined to comply with the neighborhood norms. In contrast, urban individuals have a relatively high proportion of colleagues in their social networks, and interact relatively less frequently with their neighbors in the surrounding community. Urban individuals are influenced not only by neighborhood social norms but also by group social norms, while rural individuals are more influenced by neighborhood social norms.

**Table 6:** Heterogeneity analysis: Urban–rural, gender, and life-course differences.

	Urban–rural		Gender differences		Life course difference	
	(1)	(2)	(3)	(4)	(5)	(6)
	Rural	Urban	Female	Male	Unmarried	Married
Neighborhood social norm	0.0988^***^ (4.00)	0.0739^***^ (6.03)	0.0394^***^ (2.82)	0.1100^***^ (3.20)	0.0619^***^ (2.13)	0.0832^***^ (7.22)
Group social norms	0.0006 (0.01)	0.0528^***^ (2.76)	0.0285 (1.22)	0.0401 (0.71)	−0.0680 (−0.99)	0.0513^***^ (2.90)
Control variables	YES	YES	YES	YES	YES	YES
Observations	2,246	1,075	1,557	1764	472	2,849
Provincial fixed effect	YES	YES	YES	YES	YES	YES

*T*-values in parentheses.

*** *p* < 0.01.

#### Gender differences

There are significant differences between men and women in the fertility decision-making process. On the one hand, the gender concept of “men dominate outside and women dominate inside” makes men more susceptible to social norms in traditional Chinese culture. Male social networks are more extensive than females, and men are more “face-conscious” in social activities and more interested in gaining the approval of others (Wang et al., 2015). As a result, men have a stronger desire to follow established social norms. On the other hand, women take the main responsibilities of childbearing, and the traditional gender division of labor also makes women bear more childcare pressure. Women not only face the costs of childbirth but also suffer from the “motherhood penalty” (career development and salary income will be affected due to childbirth). As a result, there is a large difference in the cost of fertility between men and women (Xing, 2020; Ma, 2022).

The overall samples were further divided by gender to further explore the differences in the influence of social norms. As shown in Table 6, models 3 and 4 show that the coefficient of male fertility intentions and neighborhood social norms was 0.1100, while that of women was 0.0394, both were significant at the 1% level, indicating that both men and women were influenced by neighborhood social norms, especially the former. In terms of group social norms, the coefficient of fertility intentions with group social norms was 0.0285 for men and 0.0401 for women, which indicated that men are more likely to be influenced by group social norms. Male fertility intentions are more influenced by social norms, as they desire to comply with existing social norms. Gender role perceptions make women bear higher costs in childbearing, women pay higher costs to follow social norms, which moderate the effect of social norms on fertility intentions.

#### Life course differences

Research has shown that, following significant life events such as marriage or childbirth, people tend to align with existing social norms to avoid cognitive dissonance, a phenomenon known as the adaptation effect (Moors, 1996). Those who have a steady date or are married have higher fertility intentions than those without a partner or who are unmarried and cohabiting (Liefbroer, 2009). Individuals with different life stages have different psychological demands for children, and as a bond between couples, children have a significant impact on stabilizing marital relationships and resolving family conflicts (Xu et al., 2013). In Chinese culture, having children after marriage is considered reasonable and desirable; thus, the social pressure to have children increases after individuals married. This pressure manifests as expectation from parents and relatives, which contributes people to have children and to conform to social norms (Baizán et al., 2003). The social normative pressures faced and felt by individuals could change significantly after life course change.

The most important life course change for fertility behavior is whether or not to marry. Therefore, we divided respondents’ life course stages according to marital status (unmarried vs. married) to further examine the influence of social norms. As shown in Table 6, models 5 and 6 showed that the coefficient of fertility intentions and neighborhood social norms for the unmarried group was 0.0619 and that of the married group was 0.0832, both were significant at the 1% level, indicating that the married group was more influenced by neighborhood social norms. Moreover, the coefficient between fertility intention and group social norms for the unmarried group was −0.0680 but insignificant, while the coefficient between fertility intention and group social norms for the married group was 0.0513, which is significantly positive at the 1% level. Therefore, the unmarried group was negatively influenced by group social norms. Unmarried individuals are generally younger, they focus more on career development, which reduces their fertility intentions. As individuals enter marriage and their life course changes, their attitudes toward social norms change and they become more willing to conform to social norms. In the Chinese social norm of marriage, it is considered proper to have children after marriage. Therefore, the blessings usually given to newlyweds include “We hope you will have a child soon” and “I hope you will have two children within three years.” Thus, many couples face pressure from parents, relatives, and colleagues to have children after marriage. After the Two-Child Policy, many couples with only one child also face pressure from social networks to have a second child or more. Social pressure drives them to have higher fertility intentions.

## Discussion

Low fertility levels are a major challenge for several countries, and China’s current low fertility rate is an issue of concern for all sectors of Chinese society. Low fertility problem not only affect countries’ population size and demographic structure, but also their future innovation and development capacity. Thus, it is important to clarify the causes of low fertility levels in China. The implementation of the OCP not only changed people’ fertility behavior but also had a profound impact on the traditional concepts and social norms related to fertility. However, the transformation of social norms is a gradual process, which is why the effects of OCP persist even years after China’s fertility policy has shifted.

We explored the influence of social norms on individuals’ fertility intentions from two perspectives: spatial proximity and social proximity. The study found that individual’s fertility intentions were influenced by social norms; both neighborhood social norms and group social norms had significant effects. The role of social norms in shaping individual fertility intentions varied by gender, *hukou*, and life course; specifically, men, rural residents, and married individuals were more significantly influenced by social norms.

There is a lack of research on the correlation between social norms and fertility intentions in the Chinese context. Our study verified that fertility intentions were significantly and positively associated with neighborhood social norms and group social norms. The fertility intention of individuals increased by 15.10% for every unit increase in the actual fertility rate of the community, and increased by 8.10% for every unit increase in the actual fertility rate of the occupation. Nie et al. (2021) found that community peer fertility can increase individual fertility intentions, with each unit increase in community peer fertility, the probability of having only one child decreased by 14.3%, whereas it increases the probability of having three children by 9.3% and four or more children by 4.8%. Using Chinese religious culture as a normative proxy, Peng (2009) concluded that clan networks increased fertility by 8%. Compared with previous studies, our study emphasized on impact of the social norms at the group level with the occupation as the reference in addition to the neighborhood social norms. Occupation is the most important social identity of an individual, and colleagues are important members of an individual social network. Therefore, the influence of occupational social norms on fertility intentions cannot be ignored. The results of the study show that there is a significant positive effect of group social norms on individuals’ fertility intentions.

We confirmed that social norms were an important factor affecting individuals’ fertility intentions and provided empirical support for the theoretical framework outlined in the preceding section. The analysis of the trends in fertility levels in China (Figure 1) revealed that they have declined significantly owing to modernization and the implementation of OCP. Modernization and its associated factors, such as economic growth, increased female education, and health, directly affect individuals’ fertility intentions. Prior to the OCP implementation, fertility levels had been steadily declining because of modernization. In 1980, China formally implemented OCP, and the restrictive fertility policy had a restraining effect on individuals’ fertility behaviors, which forcibly changed individuals’ fertility intentions and behaviors and rapidly reduced the fertility level. Simultaneously, modernization and fertility policies not only directly changed individuals’ fertility behaviors and reduced the fertility level of the whole society, but also subtly changed social norms from “having more children” to “having fewer children.” The new social norms, after they had been formed over time, would persistently affect individuals’ fertility intentions. This has been confirmed by surveys on fertility intentions, presented in Table 1, which shows that two children is already an ideal family size for the majority of people.

In contrast, modernization and fertility policies may also moderate the role of social norms on individual fertility intentions. Modernization may weaken the influence of social norms on individuals. The improved status of women, rise of individualism, modernization of fertility, and increased sense of independence brought about by modernization may weaken the influence of social norms on fertility intentions. In the urban–rural heterogeneity test, we found that urban residents were less influenced by social norms than rural ones. The degree of modernization and urbanization was generally higher in urban regions than in the rural, and urban residents had more modern concepts and a stronger sense of independence, and therefore, they were less influenced by social norms (Yang et al., 2000; Shi et al., 2022). Fertility policies, in contrast, modified the influence of social norms on fertility intentions by giving rewards or imposing restrictions for complying or not complying with the new social norms. During the past few decades, noncompliance with OCP has been punished with fines and other sanctions, and people have been forced to adopt fertility compliance behaviors (Wang, 2012; Zhang, 2017). When fertility policies were transformed into incentive fertility policies from 2015, China government implemented a series of supportive measures to promote people’s fertility intentions and increase the fertility rate. Incentive fertility policies promote people compliance with social norms through supportive incentives in the form of giving rewards. However, the social norms of low fertility that had developed under OCP policies had become an important factor in restricting people’s fertility and reducing the effect of fertility-friendly policies.

In China’s urban–rural dual system, urban and rural residents have different fertility intentions and are influenced by different social norms. China has a large urban–rural migrant population, with many migrants from rural areas living in cities for many years. Immigrant integration theory suggests that migrants are influenced by the social norms of the place they move to and adjust their fertility intentions, eventually resulting in a convergence of fertility behaviors between immigrants and local residents (Findley, 1980; Zak et al., 2002; Wang and Fan, 2012). Therefore, the fertility perceptions of rural migrants are influenced by the social norms of the residents of the cities around them, which causes urban low-fertility norms to gradually spread from urban centers to the countryside (Wang and Fan, 2012). In the context of urban–rural integration, this issue should be examined further. Therefore, it is necessary to raise the fertility intentions of the whole population in a timely and comprehensive manner and to reshape the social norms of fertility.

Gender differences in fertility intentions suggest that women have lower fertility intentions and are less positively influenced by social norms, possibly due to the higher opportunity costs of childbearing and the possibility of facing motherhood punishment. The traditional division of gender roles in China makes the involvement of fathers in the child-rearing process less frequent or even nonexistent. The excessive pressure of childcare also reduces women’s fertility intentions (Li, 2020; Xu, 2020). In the process of building a fertility-friendly environment, we must pay attention to female career-childbearing conflict, improve corresponding policies and regulations, ensure gender equality in the labor market, and protect female legitimate rights and interests in employment. Further, it is necessary to continue to explore the system of maternity leave, and paternity leave, urge men to pay attention to their responsibilities and duties as fathers, advocate for a more balanced division of labor in the family, and make fathers to participate more frequently in the family sphere.

Our research on life-course differences shows that unmarried individuals are less influenced or even negatively influenced by group social norms. Unmarried individuals comprise the main group for future fertility, and timely changes their fertility perceptions could have an important impact on future fertility levels. It has been shown that social propaganda and national media has an important role in shaping individual perceptions and values (Storey and Kaggwa, 2009). Many young people are afraid of marriage and childbearing due to the increasing negative publicity about marriage and childbearing in the current mass media. Therefore, government should pay attention to negative public opinion and provide timely guidance and correction. Public media and institutions should promote mainstream family values. In addition, in public opinion and social occasions, it is necessary to create a positive public opinion environment for fertility, to guide young people to establish a positive family concept, and to reduce worries and negative perceptions about marriage and fertility.

## Conclusion

Based on social norms theory, this paper explored the influence of neighborhood social norms and group social norms on individual fertility intentions. By using 2018 CFPS data, we found that: (i) Individuals’ fertility intentions differ across regions and occupational groups, with the highest fertility intentions being found in the western region, followed by the central and eastern regions. Additionally, farmers and business owners exhibited higher fertility intentions, while those who worked in government/institutions/research institutes/state-owned enterprises exhibited the lowest fertility intentions. (ii) Individuals’ fertility intentions were positively influenced by neighborhood social norms and group social norms, i.e., the higher the actual fertility rate within their community and occupation, the higher the fertility intentions. (iii) Rural residents and men were more influenced by social norms, compared with urban residents and women, respectively. Individual fertility intentions were influenced by social norms differently depending on their life course.

This paper improves the theoretical framework of fertility decisions by incorporating social norms factors into fertility decisions. The role of social norms in shaping individuals’ fertility intentions explains why no timely shift in fertility levels has occurred after the Chinese adjustment of its fertility policy. The persistence and inertia of the influence of low-fertility social norms on individuals’ fertility intentions should be recognized. Moreover, the influence of neighborhood social norms on fertility intention explains the regional differences in fertility levels in China, while group social norms explain the differences in fertility levels among people with different occupational backgrounds. Our study verified the role of social norms in shaping individuals’ fertility intentions. Therefore, in the process of raising fertility levels and reshaping social norms on fertility, it is important to give full play to the power of communities, groups. Publicity to encourage fertility is placed in the community and workplace to strengthen public opinion and policy guidance. At the same time, we note that compared with group social norms, neighborhood social norms play a greater role. Therefore, community can play a more effective role in creating the fertility-friendly environment society.

The present study has several limitations. First, social norms influence individuals’ perceptions and behaviors, especially through the important individuals in social networks. However, because the survey data we used did not contain information on social networks and social interactions, we were unable to test the influence of important individuals on individuals’ fertility perceptions. Second, China implemented the Universal Two-Child policy in 2016. The fertility policy adjustment may lead to changes in fertility intentions. Our research data were cross-sectional and we were unable to completely strip the influence of social norms and the new policies on fertility intentions. Bridging these limitations will the focus of our future research.

## Data availability statement

Publicly available datasets were analyzed in this study. This data can be found at: http://www.isss.pku.edu.cn/cfps/.

## Ethics statement

The studies involving human participants were reviewed and approved by Member of Biomedical Ethics Committee of Peking University. The patients/participants provided their written informed consent to participate in this study.

## Author contributions

XY and JL contributed to conception and design of the study and wrote sections of the manuscript. JL performed the statistical analysis and wrote the first draft of the manuscript. All authors contributed to the article and approved the submitted version.

## Funding

This research was funded by Major project of the Key Research Institute of Humanities and Social Sciences of the Ministry of Education in China (17JJDGJW006) and the National Social Science Fund of China (18BRK024).

## Conflict of interest

The authors declare that the research was conducted in the absence of any commercial or financial relationships that could be construed as a potential conflict of interest.

## Publisher’s note

All claims expressed in this article are solely those of the authors and do not necessarily represent those of their affiliated organizations, or those of the publisher, the editors and the reviewers. Any product that may be evaluated in this article, or claim that may be made by its manufacturer, is not guaranteed or endorsed by the publisher.

## Appendix

TABLE 7 Sub-sample test.

**Table:**

	Fertility intention	Fertility intention	Fertility intention
**Panel A: All samples**			
Neighborhood social norm	0.0891*** (9.36)		0.0882*** (9.34)
Group social norms		0.0452*** (3.32)	0.0391*** (2.92)
Control variables	YES	YES	YES
Observations	5,507	5,507	5,507
Provincial fixed effect	YES	YES	YES
**Panel B: Fertile age samples**			
Neighborhood social norm	0.0809*** (7.28)		0.0809*** (7.35)
Group social norms		0.0434** (2.42)	0.0431** (2.43)
Control variables	YES	YES	YES
Observations	3,321	3,321	3,321
Provincial fixed effect	YES	YES	YES
**Panel C: Beyond fertility age samples**			
Neighborhood social norm	0.0937*** (5.41)		0.0920*** (5.28)
Group social norms		0.0329* (1.66)	0.0193* (1.70)
Control variables	YES	YES	YES
Observations	2,186	2,186	2,186
Provincial fixed effect	YES	YES	YES

T values in parentheses; **p* < 0.1; ***p* < 0.05; ****p* < 0.01.
